# *Trypanosoma cruzi* strain TcI is associated with chronic Chagas disease in the Brazilian Amazon

**DOI:** 10.1186/1756-3305-7-267

**Published:** 2014-06-11

**Authors:** Rosa Amélia Gonçalves Santana, Laylah Kelre Costa Magalhães, Laise Kelman Costa Magalhães, Suzane Ribeiro Prestes, Marcel Gonçalves Maciel, George Allan Villarouco da Silva, Wuelton Marcelo Monteiro, Felipe Rocha de Brito, Leila Inês de Aguiar Raposo Câmara Coelho, João Marcos Barbosa-Ferreira, Jorge Augusto Oliveira Guerra, Henrique Silveira, Maria das Graças Vale Barbosa

**Affiliations:** 1 University of the State of Amazonas (Universidade do Estado do Amazonas), Manaus, Brazil; 2 Heitor Vieira Dourado Tropical Medicine Foundation (Fundação de Medicina Tropical Heitor Vieira Dourado), Manaus, Brazil; 3 Federal University of Amazonas (Universidade Federal do Amazonas), Manaus, Brazil; 4 Leônidas and Maria Deane Institute, Fiocruz of Amazon, Manaus, Brazil; 5 Francisca Mendes Hospital, Manaus, Brazil; 6 Institute of Hygiene and Tropical Medicine (Instituto de Higiene e Medicina Tropical), New University of Lisbon (Universidade Nova de Lisboa), Lisbon, Portugal

## Abstract

**Background:**

Chagas disease in the Amazon region is considered an emerging anthropozoonosis with a predominance of the discrete typing units (DTUs) TcI and TcIV. These DTUs are responsible for cases of acute disease associated with oral transmission. Chronic disease cases have been detected through serological surveys. However, the mode of transmission could not be determined, or any association of chronic disease with a specific *T. cruzi* DTU’s. The aim of this study was to characterize *Trypanosoma cruzi* in patients with chronic Chagas disease in the State of Amazonas, Brazil.

**Methods:**

Blood culture and xenodiagnosis were performed in 36 patients with positive serology for Chagas disease who participated in a serological survey performed in urban and rural areas of Manaus, Amazonas. DNA samples were extracted from the feces of triatomines used for xenodiagnosis, and the nontranscribed spacer of the mini-exon gene and the mitochondrial gene cytochrome oxidase subunit II (COII) were amplified by PCR and sequenced.

**Results:**

Blood culture and xenodiagnosis were negative in 100% of samples; however, molecular techniques revealed that in 13 out of 36 (36%) fecal samples from xenodiagnosis, *T. cruzi* was characterized as the DTU TcI, and different haplotypes were identified within the same DTU.

**Conclusion:**

The DTU TcI, which is mainly associated with acute cases of Chagas disease in the Amazon region, is also responsible for chronic infection in patients from a region in the State of Amazonas.

## Background

Chagas disease (CD) is a complex zoonosis found in South and Central America and is considered to be one of the most important neglected diseases. It is estimated that between 8 and 11 million people are infected and that over 25 million are at risk of developing the disease [1]. In Brazil, the epidemiological patterns of the disease have changed as a result of control activities and environmental, economic, and social changes. In the Amazon region, infection with *Trypanosoma cruzi* was previously thought to be an enzootic disease of wild animals [2]. However, in recent years, it has been recognized as an important emerging anthropozoonosis, with reports of acute and chronic [3-7] cases and unclear mechanisms of transmission.

The etiologic agent of CD is the flagellate protozoan *T. cruzi*, which is primarily transmitted through the feces of infected triatomines, may also be transmitted by blood transfusion, transplacental route, organ transplantation, laboratory accidents and orally through contaminated food [8]. This parasite presents high genetic variability and is divided in 6 specific discrete typing units (DTUs), termed TcI to TcVI [9,10]. Understanding the genetics and diversity of the parasite will provide information about its evolution, biological behavior, and epidemiological patterns; the natural history of infection; and issues related to the diagnosis and treatment of the disease [10,11].

In the Brazilian Amazon, Venezuela, Colombia, Central America, and North America, TcI is the prevailing DTU and is responsible for most cases of acute and chronic cardiac CD, whereas TcIV causes sporadic cases of acute CD in the Amazon [6]. In recent years, the number of chronic CD cases detected at the Tropical Medicine Foundation (*Fundação de Medicina Tropical*) outpatient clinics has increased [4,5,12].

Previous serological surveys revealed that prevalence of *T. cruzi* chronic infection in the municipalities of Coari and Tefé and also in Manaus rural areas is 0.5%, 1.9% and 1.2%, respectively [13]. Furthermore, [14] registered a 13% prevalence in the Rio Negro microregion. Comparing the range of prevalence in both studies, our study area can be considered of low endemicity.

Understanding the emergence and expansion of CD in the Amazon requires knowledge of the diversity of *T. cruzi* circulating in the region. Thus, the aim of this study was to characterize *T. cruzi* DTUs in patients with chronic infection in the State of Amazonas, Brazil.

## Methods

### Ethical issues

This project was approved by the ethics committee on human research of the Dr. Heitor Vieira Dourado Tropical Medicine Foundation (*Fundação de Medicina Tropical Dr. Heitor Vieira Dourado*) (Approval No. 1986). Patients diagnosed with CD were referred for clinical follow-up as recommended by the Brazilian Ministry of Health. All participants involved in the study voluntarily signed an informed consent form.

### Study area

**Figure 1:**
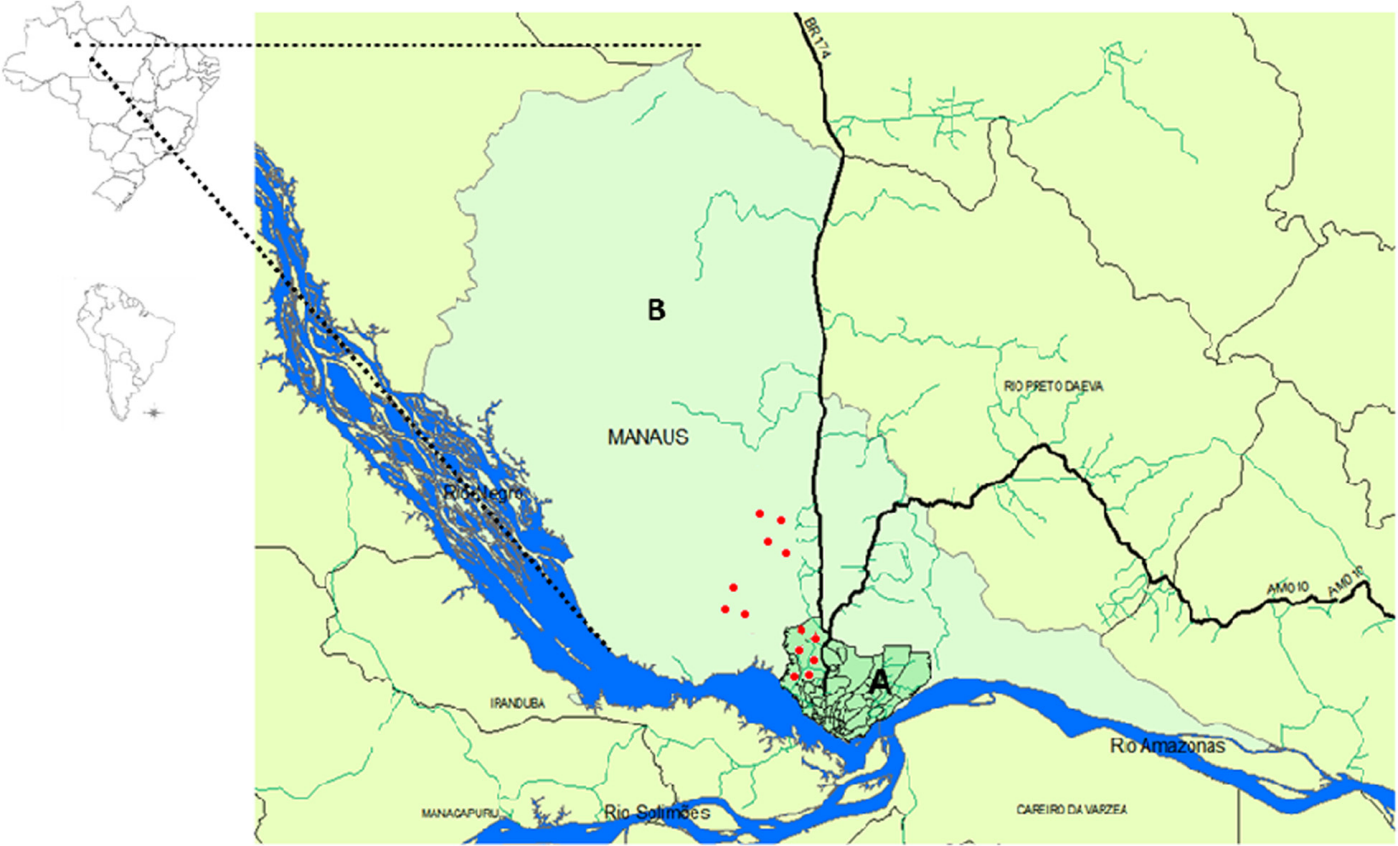
Map of Manaus highlighting the study areas (A: urban area, B: rural area) and the location of participants (red dots).

The study was performed in rural and urban areas in the west side of the city of Manaus, State of Amazonas (AM), Brazil, where autochthonous acute and chronic cases of CD [13] and the presence of infected vectors and reservoirs of *T. cruzi*[15] were reported. The area as the whole Amazon state is considered a low endemicity area for Chagas disease [3,13]. In the rural area, data collection was performed in the Tarumã Mirim settlement, which is located on federal highway BR 174. In the urban area, the study was performed in the Tarumã neighbourhood, which is partly located within an area of environmental protection and partly within an area of real estate expansion, which has caused environmental degradation (Figure 1).

### Study population

Patients included in this study were identified during a serological survey performed from October 2010 to July 2012. Of the 1837 participants evaluated during the survey, 44 were positive by at least two of the three serological tests performed: enzyme-linked immunosorbent assay (ELISA) (Wama® and Bioeasy®), indirect immunofluorescence (IIF) (Wama), and Western Blot (BioMérieux®). Of these 44 patients, 36 underwent clinical follow-up at the Dr. Heitor Viera Dourado Tropical Medicine Foundation, and whole blood culture, xenodiagnosis, and molecular techniques for the detection of *T. cruzi* in the feces of triatomine bugs used for xenodiagnosis were performed.

### Whole blood culture

Eight ml of blood were collected in heparinized tubes from patients who agreed to participate, for each patient, 100ul whole blood was distributed into 2,5 ml of NNN culture media containing rabbit blood and 40 mg/ml of gentamycin sulphate. Triplicates were made for each patient. The search for flagellate forms was performed for a period of 120 days by inverted optical microscopy [16,17].

### Xenodiagnosis

Xenodiagnosis was carried out using two cages each containing 20 III- and IV-stage nymphs of *Triatoma infestans* reared in the laboratory, that were put in contact with the patient’s arm for 30–40 min [18,19]. After the blood meal, triatomines were maintained at 27°C and after 30, 60, 90, 120, and 180 days of incubation, the feces of each insect were analyzed microscopically and harvested for molecular characterization. Feces of the 40 nymphs used for each patient were collected in pools of 10 nymphs, at each of the five time points after the blood meal, totaling 1440 pools. Although Triatominae species susceptibility for different strains of *T. cruzi*, on xenodiagnosis, may differ [20], *T. infestans* had a better performance on natural xenodiagnosis and is normally refractory to *T. cruzi*[21,22].

### DNA extraction

For DNA extraction, the technique described by Zulantay and colleagues [23] was used. There were a total of 1440 nymphs (40 nymphs from each of 36 patients). Feces of triatomines in pools of 10 triatomines of the same patient and time of collection were placed in 1.5-ml microtubes with 500 μl of phosphate-buffered saline (PBS) pH 7.2 and incubated in a water bath for 15 minutes at 98°C. Next, the samples were centrifuged for 3 minutes at 3500 rpm. A total of 200 μl of the supernatant was removed and stored at -20°C; this sample was used for PCR.

### DNA amplification of the mini-exon gene

The DNA target of the non-transcribed spacer of the mini-exon gene was amplified according to the multiplex protocol previously described by Fernandes and colleagues [24]. The amplification process was performed with an initial denaturation cycle of 95°C for 5 minutes; 35 cycles of: denaturation at 94°C for 30 seconds, annealing at 55°C for 30 seconds, and extension at 72°C for 30 seconds; and a final 10 minute extension at 72°C. The amplified products were analyzed by electrophoresis on a 2% agarose gel stained with ethidium bromide. The 150 bp product is characteristic of *T. cruzi* zymodeme (Z3) of DTUs TcIII or TcIV, 100 bp is characteristic of *T. rangeli*, 200 bp corresponds to *T. cruzi* TcI, and 250 bp is characteristic of *T. cruzi* TcII.

### Analysis of polymorphisms of the gene encoding cytochrome oxidase subunit II (COII)

All samples were subjected to mitochondrial DNA typing by analyzing polymorphisms in the COII gene. Amplifications were performed with the primers TcMit 31 and TcMit 40, which were designed to amplify a product of approximately 400 bp [25]. For each PCR reaction, 2 μl of the sample was used under the following amplification conditions: an initial denaturation cycle at 94°C for 5 min, followed by 35 cycles of 30 seconds at 94°C, 2 minutes at 48°C, and 2 minutes at 72°C, with a final extension at 72°C for 5 minutes. Positive and negative controls were included in each reaction. PCR products were visualized on a 1.5% agarose gel stained with ethidium bromide.

The amplified PCR products were purified using the Wizard SV Gel and PCR Clean-up System kit (Promega) and sequenced in both directions using the primers TcMit31 and TcMit40. The sequencing reactions were performed using 1–3 μl of the PCR product, 0.33 pmol of primer, 2.0 μl of 5× buffer, 1.0 μl of Big Dye Terminator v.3.1 CycleSequencing Kit (Applied Biosystems), and water to make a final volume of 10 μL. The reactions were performed in a Mastercycler Gradient thermocycler (Eppendorf). PCR products were purified using the Wizard SV Gel and PCR Clean-up System kit (Promega) according to the manufacturer’s recommendations. DNA sequencing was performed on a DNA Analyzer ABI 3130XL (Applied Biosystems). Nucleotide sequences were edited with the program BioEdit version 7.0.5.2. [26].

Validation of sequence quality was performed, and the consensus sequence of the sense and antisense strands was mounted and aligned with standard sequences obtained from GenBank (http://www.ncbi.nlm.nih.gov/). Generated sequences were deposited at Genbank [Genbank: KJ636060 to Genbank:KJ636072]. The following standard strains were used: TcI (Silvio ×10 cl4), TcII (Esmeraldo cl3), TcIII (M6241 cl6), TcIV (CANIII cl1), TcV (Mn cl2), and TcVI (CL Brener), with the respective accession numbers [GenBank: EU302222.1, AF359035.1, AF359032.1, AF359030.1, DQ343718.1, and DQ343645.1].

Phylogenetic trees were constructed using the neighbor-joining method included in the program MEGA 5.2 [27]. The bootstrap analysis was inferred based on 1500 replicates.

## Results

Thirty-six patients were included in the study: 21 (58%) were from a rural area while 15 were from a periurban area; 28 (78%) were originally from the State of Amazonas; 18 (50%) were male and 18 (50%) worked in agriculture. Patient age ranged between 8 and 73 years, with the highest percentage (36%) of patients aged more than 50 years (Table 1). Out of the 36 patients, 26 (72%) were reactive by ELISA + IIF, two (6%) by ELISA + Western Blot, and 8 (22%) by the three tests (ELISA + IIF + Western Blot; Table 2).

**Table 1:** **Number of patients included in the study by gender according to area of residence**, **age**, **and origin**

**Variables**	**Female**	**Male**	**Total**
**Area**			
**Rural**	10 (28%)	11 (19%)	21 (58%)
**Periurban**	8 (22%)	7 (19%)	15 (42%)
**Origin(by state)**			
**Amazonas**	18 (50%)	10 (28%)	28 (78%)
**Pará**		2 (6%)	2 (6%)
**Ceará**		3 (8%)	3 (8%)
**Maranhão**		1 (3%)	1 (3%)
**Piauí**		1 (3%)	1 (3%)
**Minas Gerais**		1 (3%)	1 (3%)
**Age group**			
**8 to 20**	2 (6%)	2 (6%)	4 (11%)
**21 to 30**	4 (11%)	0	4 (11%)
**31 to 40**	4 (11%)	5 (14%)	9 (25%)
**41 to 50**	4 (11%)	2 (6%)	6 (17%)
**> 50**	4 (11%)	9 (25%)	13 (36%)

**Table 2:** Results of PCR of samples from reactive patients for CD serology

**Serological tests**	**No. of positive patients**	**Mini-exon PCR**	**PCR(COII)**
**ELISA** **+** **IIF**	26 (72%)	1	9
**ELISA** **+** **WB**	2 (6%)	0	0
**ELISA** **+** **IIF** **+** **WB**	8 (22%)	2	4
**Total**	36 (100%)	3	13

### Blood culture and xenodiagnosis

There was no growth of *T. cruzi* in blood cultures, which may be a consequence of standard protocol used in the laboratory that uses a low amount of patient’s blood to start hemocultures, while other protocols use higher amounts of patient’s blood [20]. No flagellated forms were observed in the fresh triatomine stools.

### DNA amplification of the mini-exon gene

*T. cruzi* positivity was observed in PCR of the mini-exon gene of triatomine samples after 180 days of xenodiagnosis of three (8.3%) patients, with the profile of amplified products compatible with TcI (Table 2).

### Analysis of the mitochondrial gene COII

**Figure 2:**
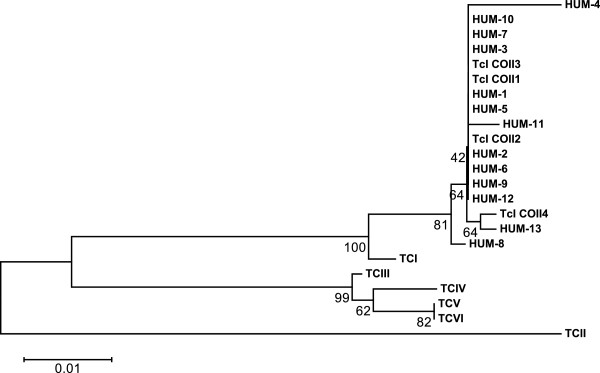
**Phylogenetic tree showing the distribution of *Trypanosoma cruzi *isolates in rural and periurban areas of Manaus, State of Amazonas, Brazil, based on the sequencing of the gene encoding cytochrome oxidase subunit II.** The following standard strains obtained from GenBank (DTU (strain name – access number)) were used: TcI (Silvio ×10 cl4 – EU302222.1), TcII (Esmeraldo cl3 – AF359035.1), TcIII (M6241 cl6 – AF359032.1), TcIV (CANIII cl1 – AF359030.1), TcV (Mn cl2 – DQ343718.1), and TcVI (CL Brener – DQ343645.1). TcCOII1, haplotype described in (6).

PCR of the gene encoding COII showed a band of 420 bp amplified in 13 (36%) samples corresponding to the 13 patients already detected by Mini-exon gene amplification (Table 2). Sequencing and phylogenetic analysis of the gene encoding the COII reference strain Silvio 10× showed that chronic infection of these patients was caused by the *T. cruzi* DTU TcI (Figure 2).

### Haplotypes

**Figure 3:**
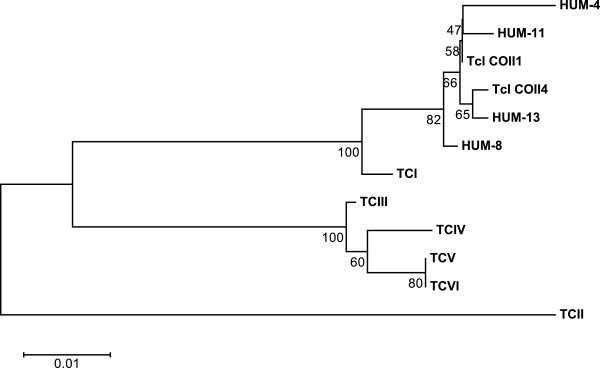
**Representation of *Trypanosoma cruzi *haplotypes identified as DTU TcI identified by sequencing of the cytochrome oxidase subunit II gene.** Standard strains obtained from GenBank [DTU (strain name – access number)]: TcI (Silvio ×10 cl4- EU302222.1), TcII (Esmeraldo cl3 -AF359035.1), TcIII (M6241 cl6- AF359032.1), TcIV (CANIII cl1 -AF359030.1), TcV (Mn cl2- DQ343718.1), and TcVI (CL Brener- DQ343645.1). TcCOII1, haplotype described in (6).

The analysis revealed the presence of four different haplotypes in the samples. Based on these sequences, haplotype TcI COII 1 [6] was the most frequent, occurring in samples of 9 patients (HUM01,02,03,05,06,07,09,10,12). The remaining sequences from the samples of four patients (HUM04 and HUM08, HUM11, and HUM13) were not previously described (Figure 3).

Of the 13 patients positive for TcI, 8 were male, their age ranged from 9 to 73 years, 9 were originally from Amazonas and live in a rural area with eating habits including the consumption of wild animal meat and palm tree fruit juice, 3 had never heard of the disease. Electrocardiogram and echocardiogram of seropositive patients show mild left ventricular diastolic dysfunction in only 2 patients. No digestive system complaints were reported.

## Discussion

This study revealed that the DTU TcI causes chronic CD in an asymptomatic group of patients in the Amazon region. This study is the first in the region to characterize TcI in patients with chronic infection, showing the insertion of man into the sylvatic cycle of *T. cruzi* and reinforcing the idea that the Amazon is an emerging area for CD [14].

TcI has a wide distribution from North America to South America. In countries of Northern South America such as Venezuela and Colombia, TcI has been reported as the predominant lineage and, association with Chagas disease pathology has been reported [28-30]. TcI has also been sporadically reported from chagasic patients throughout the southern cone. Research performed in the State of Amazonas, especially in the microregion of Rio Negro, show a disproportionate relationship between the numbers of individuals with positive anti-*T. cruzi* serology and overt clinical cases of CD, with a prevalent profile of low morbidity and mortality [3,18,31]. In this region, there are reports of seropositive patients exhibiting cardiac abnormalities with no record of digestive disorders [32].

Comparisons of the pathogenicity of the different DTUs reveal that TcI shows lower parasitemia compared with the DTUs TcV and TcVI [33]. Considering that the biological characteristics of the different DTUs play an important role in the prevalence of latent chronic Chagas infection and the low immune power of circulating *T. cruzi*, it has been hypothesized that the *T. cruzi* strains circulating in the State of Amazonas have low virulence and pathogenicity [34].

Nine out of the 13 patients characterized as DTU TcI had a common haplotype, which was also the most frequent in outbreaks reported in the Amazon by Monteiro and colleagues [6]. Eleven out of 13 had never left the Amazon region, and 4 were originally from northeastern and midwestern regions of Brasil. The *T. cruzi* haplotypes isolated from these four patients fall into the most prevalent haplotype in the region present in the study, suggesting that they acquired the infection locally, progressing to the chronic phase.

An important biological factor in the natural history and epidemiology of CD is the high diversity of vectors and wild animal reservoirs of *T. cruzi*, resulting in an intense and complex sylvatic transmission cycle. This factor may be related to parasite genetic diversity. The same study by Monteiro *et al*. [6] revealed four haplotypes of DTUs TcI and TcIV causing infections; however, no correlation between haplotype and clinical and epidemiological factors of CD or the interaction of the parasite with the vector were observed [6]. Genetic variability within TcI has been highlighted by other studies and associated with severe forms of CD in Colombia and Argentina [30,35,36]. In Venezuela, as well as other Northern South Americas, there are a number of reports showing genetically homogeneous TcI strains from humans, when compared with sylvatic TcI strains, from the same areas [30,37,38].

Although the present study did not find any association between haplotype and symptoms suggestive of CD, it is unclear whether the different clinical forms of CD may be associated with a specific *T. cruzi* DTU, clone, transmission mode or host genetics [6]. Gaining knowledge about the biological properties of the TcI lineage, which certainly plays an important role in the CD pathogenesis and response to the specific chemotherapy in the Amazon region, represents a challenge for future research [39-41].

Intensive programs of vector control and transfusion surveillance in long-established endemic areas of Brazil have been successful; however, CD has been rising in the Amazon region in the form of outbreaks associated with oral transmission. CD is thus considered as neglected and emerging, as evidenced by the occurrence of acute and chronic cases that indicate a new epidemiological profile of the disease. Thus, the importance of performing serological surveys in the region needs to be emphasized due to the cases diagnosed in the present study. Moreover, in addition to the planning and implementation of disease control measures, complementary PCR as a tool for DTU classification should also be highlighted, due to limited data on the relationship between the specific DTU genotypes/haplotypes types and clinic-epidemiological features of CD.

## Conclusions

In this study, *T. cruzi* infection was confirmed in 36 patients with reactive serology for chronic CD, parasites were detected using molecular parasitological techniques and genotyped in 13 of these patients. Furthermore, *T. cruzi* DNA was found in triatomines used for xenodiagnosis of patients with positive serology for chronic CD, being DTU TcI the detected strain.

## Competing interests

The authors declare that they have no competing interests.

## Authors’ contributions

Serological tests: LIARCC and LKCM (Laise). Parasitological tests: RAGS, MGVB, LKCM (Laylah), SRP, and FRB. Patient follow-up: JAOG, JMBBF. Molecular techniques: HS, RAGS, LKCM (Laylah), GVS and SRP. Data analysis: RAGS, MGVB, HS, MGM and WMM. Wrote the paper: RAGS, HS, and MGVB. All authors read and approved the final version of the MS.
